# Evaluating the impact of smart technology on academic eagerness, academic seriousness, and academic performance in elementary english language learners as a foreign language

**DOI:** 10.1371/journal.pone.0300147

**Published:** 2024-05-16

**Authors:** Siros Izadpanah

**Affiliations:** Department of English Language Teaching, Zanjan Branch, Islamic Azad University, Zanjan, Iran; Golestan University, ISLAMIC REPUBLIC OF IRAN

## Abstract

The proliferation of smart devices in educational settings has prompted a need to investigate their influence on learners’ attitudes and language learning outcomes. Recent advancements in smart technology (ST) have ignited curiosity regarding their impact on academic eagerness (AE), (AS), and academic performance (AP) among elementary English language learners. Despite this, there remains a dearth of comprehensive discussion in this area. This study encompasses all primary language students from the academic year 2023 as its sample. A multistage sampling method was employed for sample selection. The study introduced ST as an intervention over eight 45-minute sessions spanning two months. Data collection instruments included AE assessments adapted from Fredericks et al., an AS questionnaire developed by the researchers, and an AP questionnaire designed by Pham and Taylor. Data analysis incorporated statistical tests such as the Kolmogorov-Smirnov test, Levene test, and univariate analysis of covariance. The findings yield valuable insights into the impact of ST on AE, AS, and AP, shedding light on its potential advantages and limitations in language learning. Notably, the experimental group (EG) outperformed the control group (CG). These results contribute to the growing body of knowledge concerning ST integration in education and its consequences on AP and learner attitudes. Ultimately, this research aims to provide evidence-based recommendations for enhancing language learning outcomes and experiences among elementary English as a Foreign Language (EFL) students in the digital education era.

## Introduction

In recent years, ST has become increasingly prevalent in educational settings, transforming the way students engage with learning materials and interact with their instructors [1]. New options for language learning are made possible by the use of smart devices like tablets, smartphones, and interactive whiteboards, notably in the area of teaching English as a Foreign Language (EFL). With the potential to enhance student engagement and motivation, ST holds promise for improving academic outcomes [2–4]. The purpose of this study is to look into how ST affects AE, AS, and AP among elementary EFL learners. Hence, paying heed to English teaching is one of the significant elements of an appropriate pedagogical system [5–8].

The main goal of the teachers should be to motivate learners and enable them to get the best level of language proficiency [2,9]. ST was founded in (1987) by Martin and Knowlton. In (1991), they announced it communicating whiteboard and was regarded as the "SMART Board". In (2003), they advanced and later patented DViT (Digital Vision Touch) technology which was a vital characteristic of the SMART board. The conceptual framework in ST offers an initial point for academics and experts to further scrutinize information technology (IT) domination practices. For scholars, the framework illuminates the decisive factors of IT domination, scopes of IT governance, and impacts through anticipated relations.

Considering what has been mentioned thus far by merging IT and programs, ST is a novel educational strategy that will fundamentally alter how instruction and learning are carried out [10–15]. This method considers the role of the teacher as a guide and not a transmitter of knowledge and the role of the learner as an active, creative, critical, and participatory member, instead of a passive and consuming member [16–19].

Today, humans benefit from new technologies such as Windows Mobiles and Tablets that connect to high-speed Internet or similar. If traditional methods (lectures) are used in the classroom, the interaction between people is reduced, and teachers are forced to convey the content in the short time they have [19,20]. At the same time, regardless of the importance of the subject matter, the amount of attention continuously decreases after about 15 to 20 minutes [18].

AE refers to students’ enthusiasm, interest, and motivation toward language learning activities. AS encompasses their dedication, responsibility, and commitment to their academic tasks. AP, on the other hand, evaluates students’ language proficiency, achievement, and overall academic outcomes.

Understanding how ST influences these critical aspects of learning can provide valuable insights into optimizing instructional approaches for elementary EFL learners [21]. Prior studies have shown that integrating technology has a favorable effect on a range of educational outcomes, including student engagement, motivation, and academic accomplishment. For instance, interactive multimedia platforms, language learning applications, and online resources have been shown to increase student engagement and motivation in language learning settings [22–24].

The results of this study will offer evidence-based insights into the advantages and disadvantages of using ST in elementary language learning classes. The project will also add to the body of evidence on the use of technology in education and advise educators, policymakers, and curriculum designers about the best ways to use ST to improve language learning outcomes and experiences. On the other hand, Iran’s educational system places less focus on teaching foreign languages and cultures, and it is clear that learners do not have enough English language proficiency even after they graduate from high school. In accordance with [25]. Iranian students have limited English language proficiency, and the greater part of them study English purely for exam purposes. In addition, education professionals are eager to incorporate these technologies into the learning and teaching processes, particularly interactive technology, given the fast advancement of technology and its extensive scope of applications.

In summary, this study aims to investigate how ST affects AE, AS, and AP among basic EFL learners. The goal of this research is to shed light on the efficacy of integrating ST in language learning environments, offering useful insights to improve instructional practices and foster successful results for elementary EFL students in the digital age.

The utilization and implementation of advanced and contemporary technology have a dual effect: firstly, they enhance the process of teaching and learning for both educators and students and secondly, they enable teachers and students to leverage the potential of the World Wide Web to elevate their scientific knowledge and contribute to national development. Based on the observed instances, the ensuing research hypotheses can be formulated as follows:

Hypothesis 1: ST significantly affects the elementary level of EFL learners’ AE.
Hypothesis 2: ST significantly affects the elementary level of EFL learners’ AS.
Hypothesis 3: ST significantly affects the elementary level of EFL learners’ AP.

## Review of literature

The integration of ST in educational settings has gained significant attention in recent years, with numerous studies exploring its impact on various aspects of learning [26–28] Nonetheless, the literature has given limited focus to investigating the precise impacts of ST on AE, ASE, and AP within the context of elementary English as a Foreign Language (EFL) learners. This segment offers an overview of pertinent research studies that have explored the correlation between ST and these specific variables. By examining these studies, a thorough comprehension of the current research landscape can be established.

### Academic eagerness

Several studies have focused on the effect of technology integration on student engagement and motivation, which are key components of AE. [29] investigated the impact of mobile technology on student motivation in language learning and found that mobile applications and interactive activities enhanced students’ engagement and motivation. Similarly, [30] explored the effects of a mobile learning initiative on student engagement and reported positive outcomes, with students expressing increased motivation and interest in the language learning process. These findings suggest that ST has the potential to positively influence AE among elementary EFL learners.

The theoretical background of AE is to have a higher focal point on learning matters and topics, avoid presenting maladaptive activities and act better than other students in tests. With the advent and development of IT, the global development trend expanding more rapidly with a focus on the information element [31–33]. This started in the military environment and has been transferred to the academic and civil environments. For more than two decades, the IT system, the educational system, and the educational environment have been encompassing [34–36].

One of the most significant components in an education setting is eagerness, and researchers accentuated its role in AP compared to other individual and psychological factors. Numerous studies demonstrate that eagerness improves AP [22,37] According to [38], eagerness has a positive impact on academic success. As reported by [39], employing more interesting materials may increase students’ eagerness.

The emergence of IT in the field of education has led to significant advancements, which in turn have paved the way for the integration of network technology, replacing conventional learning methods in recent times [40]. Concerning this, there has been an increasing interest in some of the e-learning platforms and also to attract more attention, various educational institutions fully exploit e-learning. Furthermore, according to a conducted study in the United States, 73 percent of Americans assume that one of the crucial elements that lead to a nation’s accomplishment is investing in innovation and technology in the field of education [41].

The adoption of the smartboard or interactive whiteboard (IWB) in English classes is an innovative technological resource that significantly impacts students’ achievements [42]. This technology enables various applications, such as whole-class instruction using online tools, the creation of digital flipcharts, the utilization of video clips to illustrate concepts, and the ability to revise materials rapidly and extensively. These capabilities highlight the versatility and potential of the IWB as a valuable tool in enhancing the teaching and learning experience [43]. In [44] study, the author examines the utilization of essential tools that enable the effective utilization of smartboards. These tools encompass CD-ROMs, presentation software, spreadsheets, internet pages, websites, and audio-visual materials accessed directly from the computer connected to the smartboard. Several studies on schools in the United States context have revealed that smartboards have a beneficial impact on language learning [45–48].

Considering what has been mentioned thus far, to date, there is a scarcity of pragmatic studies that critically evaluate the practicality of intelligent technology in classroom environments. Most of the research conducted in this domain has primarily adopted an exploratory perspective, relying on methods such as observations and qualitative interviews with both learners and instructors [49]. Previous research indicates that skill development is a time-consuming and challenging process that cannot be fully accomplished within a brief experiment. However, there has been a lack of emphasis on the long-term impact of intelligent technology, and uncertainties persist regarding its sustained effect on specific skills over an extended period [8,50].

### Academic seriousness

Regarding AS, studies have examined how technology integration can foster students’ dedication, responsibility, and commitment to their academic tasks [51]. [52] conducted a study that integrated technology into the English classroom and found that it enhanced students’ sense of responsibility and self-directed learning. They observed that students took ownership of their learning process and were motivated to complete tasks independently. In their study, [53] investigated the influence of online peer feedback on students’ writing performance and found that it had a positive effect on enhancing students’ dedication to revising and editing their written assignments. These findings highlight the potential of ST to enhance AS among elementary EFL learners.

AS is a psychological concept that stems from an intrinsic eagerness that can also be referred to as AE [52]. AS is a skill that empowers individuals to establish specific objectives and subsequently attain them. AS is understanding and learning capacities and the effort to realize them. Therefore, by creating eagerness, the seriousness of education can be improved in learners [54,55]. AS leads to academic self-fulfillment [56,57] influences different types of educational activities, and causes a person’s desire to achieve educational goals.

### Academic performance

Regarding AP, prior studies have examined the impacts of integrating technology on language proficiency, academic achievement, and overall educational outcomes. A study by [12] explored the impact of a mobile-assisted language learning program on elementary EFL students’ vocabulary acquisition. The findings revealed significant improvements in students’ vocabulary knowledge, suggesting that ST can enhance language proficiency. Additionally, a study by [47] investigated the effects of computer-mediated communication on students’ oral proficiency in a language-learning context. The results demonstrated significant gains in students’ speaking skills, further supporting the positive influence of technology on AP.

Information and knowledge are the most basic parts of human beings, societies, and nations [58]. It is one of the most important achievements of technology development is the change in the field of education. Hence, the effectiveness and success of an educational system can be measured by the high level of AP demonstrated by its students across various courses [12,53]. Over the years, researchers have used academic intelligence to explain the factors affecting performance. However, many recent studies show that intelligence alone is not a reliable predictor of student AP [59,60]. Students’ AP in a school also depends on the student’s basic abilities and current learning processes. In the comprehensive models of student’s AP provided by researchers, the main factors affecting AP have been identified as student cognitive and eagerness characteristics, family abilities and facilities, and common teaching and learning methods in school.

One of the individual factors affecting AP is school intelligence [61,62]. The application of new technologies offers new solutions in the development of the educational system, the results of which are the establishment of smart schools (Batool et al., 2023). ST comprises interconnected elements strategically designed to foster students’ curiosity and promote their active engagement, effectively addressing individual educational requirements. By integrating these components within a comprehensive learning environment, ST aims to enhance the educational experience [63]. In smart schools, by using e-learning in person and maintaining the school, the teacher and the student with an intelligent system try to provide services to them.

A key aspect of smart schools is to empower students to cultivate their independent thinking and creativity while leveraging the skills and expertise of educators, teachers, and parents. This collaborative atmosphere not only enhances education but also fosters a conducive learning environment that motivates students [64]. Making schools smarter not only increases the efficiency of classrooms but also helps students learn by using educational clips and various software; Beyond the auditory dimension, the visual aspect plays a crucial role in enhancing education. Smart schools capitalize on flexible curricula, allowing for innovative teaching approaches, diverse instructional methods, and a student-centered focus [65]. Overall, the literature reviewed demonstrates the potential benefits of ST integration in promoting AE, AS, and AP among elementary EFL learners. Mobile applications, interactive activities, online peer feedback, and computer-mediated communication have been shown to enhance engagement, motivation, responsibility, language proficiency, and achievement. Nevertheless, it is crucial to acknowledge that despite the valuable insights offered by previous studies, there is still a lack of in-depth exploration regarding the precise effects of ST on AE, AS, and AP within the context of elementary English as a Foreign Language (EFL) learners.

The objective of this present study is to bridge this research gap by conducting a systematic investigation into the influence of ST on AE, AS, and AP among elementary EFL learners. By adopting a quantitative research design and employing validated measurement instruments, this study aims to provide a comprehensive understanding of how ST impacts these variables. The findings of this study will contribute to the existing literature and offer valuable insights for educational practitioners seeking effective strategies to integrate ST in language learning classrooms.

## Method

### Design of study

In 2023, a quasi-experimental research study was conducted in Tabriz, a city situated in northwest Iran. The research involved a total of 76 participants who were enrolled in English language programs at various institutes. These participants were stratified and subsequently divided into two groups: The Experimental Group (EG) and the Control Group (CG).

To streamline the presentation of participants’ characteristics, a comprehensive explanation has been written, enhancing the accessibility and coherence of this information.

### Participants and sampling

The current study encompasses a sample size of 5800 elementary language learners. The inclusion criteria for participants were identified as being female, possessing an elementary level of English language proficiency as determined by Oxford’s quick placement test, and having access to ST within their respective institutes. Individuals who declined to participate or chose to discontinue their involvement in the study were excluded from the analysis.

In this study, the samples were selected using the multistage sampling method. There are 112 English language institutes in Tabriz, but only four institutes were equipped with ST for teaching and met the criteria for including the study. In the first stage, one institute equipped with ST and one institute without ST were selected using a simple random sampling method. One institute was considered the CG, and the other was the intervention group. In the second stage, one class of 38 elementary-level learners was randomly selected from each institute. With this account, the total sample size was 76 learners (38 people in each group).

### Intervention and procedure

**Fig 1:**
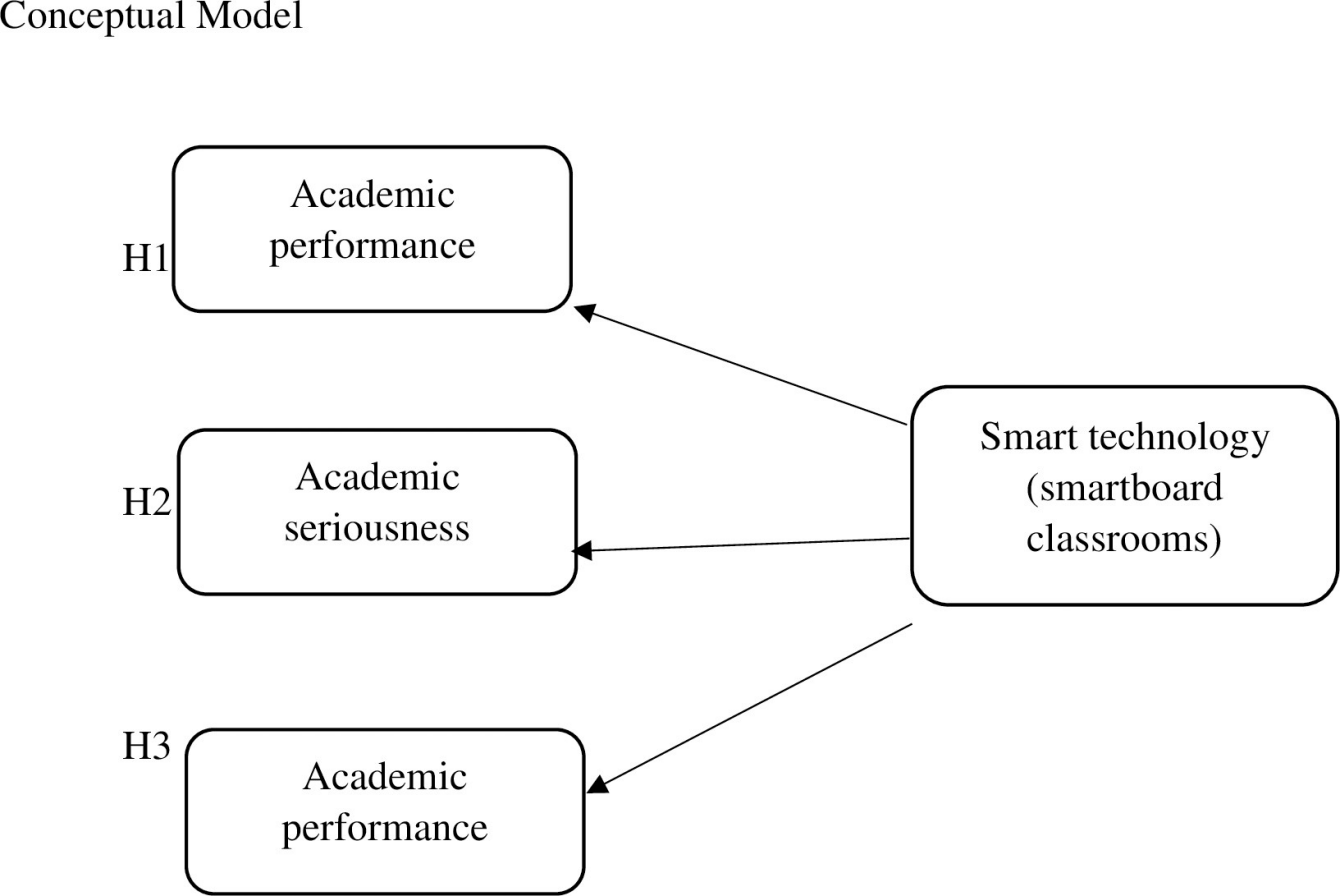
Conceptual model of research.

The intervention was using ST held in 8 sessions of 45 minutes for two months. The effect of ST-based education on AE, AS, and AP among elementary-level learners was tested in two periods, before intervention and after the last session. The CG received education without using ST (Fig 1).

### Materials and instruments

Quantitative data on AE, AS, and AP were collected using validated questionnaires. These questionnaires were designed based on existing scales that measure students’ motivation, interest, enthusiasm, dedication, responsibility, and commitment to academic tasks.

#### Academic eagerness

In this study, AE is assessed through a standardized questionnaire developed by [66] consisting of 15 questions. The questionnaire encompasses three subscales: behavioral passion (questions 1, 2, 3, and 4), emotional passion (questions 5, 6, 7, 8, 9, and 10), and cognitive passion (questions 11, 12, 13, 14, and 15). Participants rate their responses on a scale ranging from "never" (coded as 1) to "always" (coded as 5) for each question.

To establish the reliability of the AE scale, it was administered to 200 students from various medical science fields. The Cronbach’s alpha coefficient was calculated, resulting in a value of 0.66. In the original study by [66] the reliability coefficient for this scale was reported as 0.86.

### Academic seriousness questionnaire

The questionnaire utilized in this study was specifically developed by the researchers to align with the objectives of the research. It comprises 12 items that are assessed using a Likert scale. The content validity of the questionnaire was carefully examined and verified by professors and experts in the field. To assess the reliability of the questionnaire, a pilot group of 20 individuals participated, and their responses were analyzed. The calculated Cronbach’s alpha coefficient for this group was 0.890, indicating high internal consistency.

### AP questionnaire

The questionnaire utilized in this study is based on the [67] questionnaire, which comprises 48 questions. The questionnaire employs a Likert scale ranging from "very high" with a code of 5 to "none" with a code of 0. The total score range for the questionnaire is from 48 (lowest score) to 240 (highest score).

The content validity of the questionnaire was established and verified by experts and specialists, as confirmed by [68]. Furthermore, [68] conducted a reliability analysis using Cronbach’s alpha coefficient, resulting in a coefficient of 0.82, indicating good internal consistency.

### Validity of AS questionnaire

The assessment of content validity for the AS questionnaire was conducted employing two widely recognized methods: The Content Validity Index (CVI) and the Content Validity Ratio (CVR), following established reporting practices in authoritative journals of applied linguistics.

To ascertain content validity, the input of 20 experts in the field was sought. These experts were tasked with evaluating the AS questionnaire. Specifically, they were asked to determine the relevance and appropriateness of each question in the questionnaire.

The analysis of their feedback yielded the following key results:

CVR Index: The CVR index, which indicates the essentiality of each question in the questionnaire, was assessed. To establish satisfactory content validity, the CVR index should exceed a threshold value of 0.42. The results showed that all questions in the AS questionnaire exceeded this threshold, with values ranging from 0.1 to 0.8.

CVI Score: The CVI score, which assesses the clarity and relevance of each question, was also examined. To ensure strong content validity, the CVI should be higher than 0.79. In this study, all questions surpassed this threshold, with CVI values ranging from 0.60 to 0.95.

This part summarizes the specific CVI and CVR values for each individual question within the AS questionnaire. Additionally, it presents the overall CVR and CVI values for the entire questionnaire.

Notably, the overall CVR value for the entire AS questionnaire was 0.7, and the CVI value was 0.9. These values comfortably exceed the predefined threshold requirements of 0.42 and 0.79, respectively. Consequently, these findings confirm the strong content validity of the AS questionnaire, indicating that it effectively captures the intended construct of academic seriousness.

### Reliability of research questionnaires (Cronbach’s alpha)

The reliability of the research questionnaires was assessed using Cronbach’s alpha coefficient. The findings indicated that the AE questionnaire had a Cronbach’s alpha of 0.732, AS questionnaire had a Cronbach’s alpha of 0.921, and the AP questionnaire had a Cronbach’s alpha of 0.818. These coefficients were all above the threshold of 0.7, indicating that the research variables possess the required level of reliability.

### Statistical tests

For data analysis in this study, descriptive statistics employed central indicators and dispersion measures such as mean and standard deviation. In terms of inferential statistics, univariate analysis of covariance (ANCOVA) was utilized.

### Data analysis

Following the data collection phase, the collected research data was subjected to analysis using SPSS 25 software. To examine the relationships between variables, univariate analysis of covariance (ANCOVA) was employed as the statistical method for analysis.

### Oxford quick placement test

To assess the proficiency level of the students and ensure the formation of a homogeneous group, the Oxford Quick Placement Test (2007) was employed to gather relevant information.

### Data collection

To investigate the effect of ST on AE, AS, and AP among elementary EFL learners, a comprehensive data collection plan was implemented. The data collection process involved gathering quantitative data to provide a robust understanding of the research variables.

#### Informed consent

Written informed consent was obtained from all subjects before the study. There is no ethical or conflict of interest in this research. All the participants filled out consent forms.

#### Ethics approval

Our research primarily aimed at evaluating educational methods or practices rather than testing new medical interventions or substances, the potential risks to the participants were minimal or nonexistent. seeking formal approval from an ethics review board may not have been deemed necessary due to the low-risk nature of the study.

## Results

### Descriptive findings

In this section, researchers initially analyze the central indicators (mean) and dispersion (standard deviation) of the research variables:

The results of Table 1 showed.

**Table 1:** Central indicators and post-test dispersion in control and experimental groups.

		Examination Group				Control Group			
		Mean	Std. Deviation	Minimum	Maximum	Mean	Std. Deviation	Minimum	Maximum
Pretest	AE	38.553	5.285	33	58	37.711	5.881	27	50
	AS	36.158	3.175	31	43	36.500	4.373	29	44
	AP	233.061	11.893	212	254	232.763	14.652	208	255
Post-test	AE	50.500	3.769	42	58	40.290	5.392	31	50
	AS	42.000	3.734	35	48	37.895	4.118	29	45
	AP	256.895	8.895	237	275	239.921	12.562	215	257

In the pre-test, the EG exhibited a mean score of 38.553 ± 5.285 for AE, while the CG had a mean score of 37.711 ± 5.881. In the post-test, the EG displayed a mean score of 50.500 ± 3.769 for AE, whereas the CG had a mean score of 40.290 ± 5.392. These findings indicate that the EG demonstrated an increase in scores for the AE variable following the intervention.

In the pre-test, the EG had a mean score of 36.158 ± 3.175 for AS, while the CG had a mean score of 36.500 ± 4.373. In the post-test, the EG exhibited a mean score of 42.700 ± 3.734 for AS, whereas the CG had a mean score of 37.895 ± 4.118. These results indicate an increase in the AS scores for the EG following the intervention.

In the pre-test, the EG had a mean score of 233.061 ± 11.893 for AP, while the CG had a mean score of 232.763 ± 14.652. In the post-test, the EG displayed a mean score of 256.895 ± 8.895 for AP, whereas the CG had a mean score of 239.921 ± 12.562. These findings indicate that the EG experienced an increase in scores for the AP variable following the intervention.

### Inferential analysis

The research hypotheses were tested using multivariate analysis of covariance (MANCOVA). This analysis allows for a comprehensive examination of the means of one or more groups and the estimation of one or more independent variables while accounting for the influence of intervening variables, or covariates, by excluding them from the equation process. Covariance analysis is a powerful method that helps control for potential confounding factors and provides a more accurate assessment of the relationship between variables.

### Assumptions of analysis of covariance

Before analyzing the research, data using Analysis of Covariance (ANCOVA), the assumptions of the test were assessed. These assumptions include the normality of the data and the homogeneity of variance. The results of these assessments are presented in the tables below.

The assumptions of normality and homogeneity of variables were examined using the Kolmogorov-Smirnov test for normality and Levene’s test for homogeneity of variance. The results of these tests are presented below for Default 1 and Default 2, respectively:

Based on the findings presented in Table 2, the hypothesis of normality for the research variables in both the CG and EGs was supported, as indicated by the results of the Kolmogorov-Smirnov test (Sig > 0.05). Additionally, the hypothesis of homogeneity of variances was also confirmed, as indicated by the results of Levene’s test (Sig > 0.05) shown in Table 2.

**Table 2:** Data normality test and variance homogeneity.

		One-Sample Kolmogorov-Smirnov Test				Test of Homogeneity of Variances			
		Experimental Group		Control Group					
Variables		Test Statistic	Asymp. Sig. (2-tailed)	Test Statistic	Asymp. Sig. (2-tailed)	Levene Statistic	df1	df2	Sig
AE	Pretest	.115	0.196	.099	0.200*	0.814	1	74	0.370
	Post-test	.132	0.094	.109	0.200*	2.447	1	74	0.121
AS	Pretest	.105	0.200*	.111	0.200*	2.952	1	74	0.090
	Post-test	.131	0.097	.114	0.197	3.123	1	74	0.074
AP	Pretest	.119	0.192	.131	0.098	0.349	1	74	0.557
	Post-test	.102	0.200*	.106	0.200*	3.044	1	74	0.076

Assumptions 4 and 5, which include regression slope homogeneity and confirmation of the effect of the auxiliary variable, were evaluated in the analysis of covariance (ANCOVA). The results of these assessments are presented in Table 3. Before proceeding with the analysis of the research data, the assumptions of the ANCOVA test, namely the normality of the data and homogeneity of variance, were examined. The findings of these assessments are presented in the tables labeled Default 1 and Default 2, respectively. The normality of the data was assessed using the Kolmogorov-Smirnov test, while the homogeneity of variance was examined using Levene’s test. The specific results of these tests are as follows.

**Table 3:** Reception of homogeneous regression slope.

	Reception of Homogeneous Regression slope			Correlation pretest & posttest		
	variable	F	Sig	variable	F	Sig
Hypothesis1	AE *group	0.926	0.276	AE	15.418	0.001
Hypothesis2	AS *group	.341	0.685	AS	17.251	0.001
Hypothesis3	AP *group	1.823	0.135	AP	14.081	0.001

Based on the results presented in Table 3, the assumption of homogeneity of regression slope was supported by the analysis of covariance (Sig > 0.05). This indicates that the relationship between the independent variable and the dependent variable is consistent across different levels of the covariate. Furthermore, the selection of the auxiliary variable (pre-test) as a covariate in this study was confirmed (Sig < 0.05), suggesting that the inclusion of the pre-test scores as a covariate is appropriate and necessary to control for its potential influence on the dependent variable.

#### Investigation of research hypotheses

Hypothesis 1 examines the significant impact of ST on the AP of elementary-level language learners. To test this hypothesis, the researchers utilized an analysis of covariance. Before conducting the analysis, the researchers ensured that the necessary assumptions for this statistical test were met and found them to be valid. The results of the analysis of covariance are presented in the subsequent tables.

As depicted in Table 4, the use of ST (smartboard classrooms) exhibits a significant impact on AP (p = 0.001, F = 102.598). Consequently, it can be concluded that there is a significant difference in the mean scores between the two groups in the post-test, after controlling for pre-test scores. Examination of the tables reveals that the mean score of AS in the CG was 36,500 in the pre-test and 37,895 in the post-test. In contrast, the mean score of AS in the EG was 38.553 in the pre-test and 50,500 in the post-test. The significant difference between the post-test scores of the CG and EGs indicates that the implementation of the ST approach (classes equipped with smart boards) led to an increase in AE scores, independent of the pre-test factor (covariate). The effect size, represented by the ETA quadratic coefficient, indicates that 58% of the variability in AE within the EG can be attributed to the use of ST (classes equipped with smart boards).

Hypothesis 2: ST (smartboard classrooms) significantly affects AS in elementary language learners.

**Table 4:** Results of analysis of covariance for the variable of AE.

		Mean		Analysis Covariance					
Variable		Experimental	Control	Type III Sum of Squares	df	Mean Square	F	Sig	Partial Eta Squared
AE	Pretest	38.553	36.500	1858.127	1	1858.127	102.598	0.001	0.584
	Post-test	50.500	37.895						

The hypothesis was tested using an analysis of covariance. The required assumptions for this analysis were assessed and found to be valid. The results of the covariance analysis are presented in the following tables.

As depicted in Table 5, the utilization of ST (smartboard classrooms) has a significant impact on AS (Sig = 0.001, F = 21.145). This finding suggests that there is a notable difference in the mean scores of the two groups in the post-test, after accounting for the pre-test scores. Examining the tables, it is evident that the CG’s mean score for AS was 36,500 in the pre-test and 37,895 in the post-test, while the EG exhibited a mean score of 36,158 in the pre-test and 42,000 in the post-test for this variable. The significant disparity in the post-test scores between CG and EGs implies that the implementation of the ST approach (utilizing smart boards in classrooms) contributed to an increase in AS scores. The effect size, represented by the ETA quadratic coefficient, indicates that approximately 27% of the variability in AS within the experimental group can be attributed to the use of ST.

**Table 5:** The result of the study of covariance for the variable of AS Tests of between-subjects effects.

		Mean		Analysis Covariance					
Variable		Experimental	Control	Type III Sum of Squares	df	Mean Square	F	Sig	Partial Eta Squared
AS	Pretest	36.158	36.500	343.956	1	343.956	27.145	0.000	.271
	Post-test	42.000	37.895

Hypothesis 3: ST (classrooms equipped with smart boards) significantly affects AP in primary school students.

The hypothesis was tested using an analysis of covariance, and the assumptions required for this analysis were carefully examined and found to be valid. The results of the covariance analysis are presented in the tables below.

As indicated in Table 6, the use of ST (smartboard classrooms) has a significant impact on AP (Sig = 0.001, F = 53.745). This suggests that there is a significant difference in the mean scores between the two groups in the post-test, after adjusting for the pre-test scores. Examining the data in the tables reveals that the mean AP score in the CG was 232.736 in the pre-test and 239.921 in the post-test. On the other hand, the mean AP score in the EG was 233.061 in the pre-test and 256.895 in the post-test. The significant difference in the post-test scores between the CG and EGs indicates that the implementation of the ST approach (classes equipped with smart boards) while accounting for the pre-test covariate, leads to an increase in AP scores. Furthermore, the effect size of the ETA quadratic coefficient indicates that 42% of the variability in AP scores among the EG can be attributed to the use of ST (classes equipped with smart boards).

**Table 6:** Results of analysis of covariance for AP variable.

		Mean		Analysis Covariance					
Variable		Experimental	Control	Type III Sum of Squares	df	Mean Square	F	Sig	Partial Eta Squared
AP	Pretest	233.061	232.763	5410.446	1	5410.446	53.745	0.000	.424
	Post-test	256.895	239.921						

## Discussion

The main objective of this study was to investigate the impact of ST on AE, AS, and AP in elementary EFL learners. Our hypotheses were as follows:

Hypothesis 1: ST (smartboard classrooms) significantly affects AE in EFL elementary-level learners.
Hypothesis 2: ST (smartboard classrooms) significantly affects AS in EFL elementary language learners.
Hypothesis 3: ST (smartboard classrooms) significantly affects AP in EFL elementary-level learners.

Regarding the first hypothesis:

The hypothesis driving this study postulates that there is an effect of (ST on AE among elementary English as a Foreign Language (EFL) learners. AE represents the enthusiasm, interest, and motivation of students toward language learning activities. ST encompasses a wide array of digital tools and devices, such as tablets, smartphones, and interactive whiteboards, offering novel opportunities for engagement and interaction within the classroom setting.

The findings in this study correspond with previous research examining the influence of ST on students’ enthusiasm, as evidenced in studies conducted by [11,12,18,31,35,36]. These studies collectively provide a foundation for the hypothesis, suggesting that technology integration indeed has an impact on student motivation and engagement.

Nonetheless, it is important to acknowledge that while there is a broad consensus on the positive effects of ST on student enthusiasm, there are variations in the extent and nature of these effects across studies. These differences may be attributed to various factors, including the specific technologies used, the pedagogical approaches employed, and the unique contexts of each study.

For instance, findings from [55] emphasize that mobile technology and interactive activities can significantly enhance student engagement and motivation in language learning. The incorporation of ST into language learning activities can potentially make the learning process more exciting, interactive, and relevant, thus cultivating a sense of eagerness and enthusiasm toward academic tasks.

ST has the potential to offer students dynamic and interactive learning experiences. Features such as multimedia elements, interactive applications, and gamified learning platforms can render language learning more enjoyable and engaging for elementary EFL learners. The adaptability of such technology allows for personalized learning experiences that cater to individual preferences, learning styles, and interests. The interactive nature of ST may also empower students, as they actively participate, explore, and take ownership of their learning process.

Furthermore, the accessibility of ST can expand students’ exposure to authentic language materials, cultural resources, and real-world communication. The vast array of online resources and language learning applications available enables students to access diverse and pertinent content that aligns with their interests and goals. This exposure can spark curiosity, deepen engagement, and boost eagerness to explore and learn more about the English language and culture.

However, it is crucial to consider potential challenges or limitations related to ST in the context of AE. Factors such as students’ technological literacy, access to resources, and digital divide issues may influence the extent to which ST enhances AE. In some cases, the novelty of technology integration may initially generate excitement, but this enthusiasm may diminish over time if the instructional design and implementation of ST do not sustain students’ interest and motivation.

In conclusion, while our findings align with the existing literature regarding the positive influence of ST on AE among elementary EFL learners, it is imperative to recognize that our study also contributes by shedding light on the unique nuances and potential discrepancies within this context. ST integration indeed holds the potential to enhance student engagement, motivation, and enthusiasm for academic tasks. However, the specific conditions and variables at play in our study, including technological literacy and instructional design, might have influenced the extent of this impact. By examining these variations, we hope to offer valuable insights that contribute to the broader understanding of the relationship between ST and AE among elementary EFL learners.

Regarding the second hypothesis:

The hypothesis of this study posits that the utilization of ST has a discernible impact on AS in elementary English as a Foreign Language (EFL) learners. AS encompasses students’ dedication, responsibility, and commitment to their academic tasks, while ST refers to various digital tools and devices, such as tablets, smartphones, and interactive whiteboards, that offer new opportunities for engagement and interaction in the classroom.

Our findings resonate with prior research conducted by [6,17,29,54,56,63]. These studies collectively endorse the notion that ST can indeed impact AS in educational settings.

Additionally, our results align with the body of research represented by [17,69–71] which have consistently demonstrated a positive association between AS and academic self-fulfillment. This convergence of findings suggests that AS, characterized by students’ dedication, responsibility, and commitment to their academic tasks, is influenced by various factors, including the integration of ST.

While research specifically exploring the relationship between ST and AS among elementary EFL learners is limited, extant studies provide insights into the potential impact of technology integration on students’ dedication and commitment to their academic responsibilities. [3], for example, found that technology integration in English classrooms enhanced students’ sense of responsibility and self-directed learning. Their observations revealed that students took ownership of their learning process and were motivated to complete tasks independently. This evidence underscores the role of ST in facilitating students’ engagement and seriousness toward their academic obligations.

The interactive and multimedia features inherent in ST can create engaging learning experiences that capture students’ attention and enhance their focus on academic tasks. The utilization of educational apps, interactive quizzes, and multimedia resources can render learning more enjoyable, interactive, and relevant. By incorporating gamified elements, real-time feedback, and immediate access to information, ST can effectively promote a sense of purpose and seriousness among elementary EFL learners.

Nevertheless, it is crucial to acknowledge potential obstacles and limitations concerning the impact of ST on AS. Students’ varying levels of technological literacy, access to resources, and issues related to the digital divide may impact their ability to engage effectively with ST tools. Moreover, the integration of ST should be thoughtfully aligned with the curriculum and instructional objectives to ensure that it genuinely supports meaningful learning and promotes AS, rather than becoming a mere distraction or source of entertainment.

The role of teachers in supporting AS is of paramount importance when incorporating ST into instruction. Teachers can provide guidance, set clear expectations, and establish routines that promote responsibility and commitment to academic tasks. They can also leverage ST to offer timely and constructive feedback, monitor student progress, and encourage reflective practices. By fostering a supportive and motivating classroom environment, teachers can enhance students’ sense of seriousness toward their academic responsibilities.

In essence, while our findings corroborate existing research regarding the positive relationship between ST and AS, it is imperative to recognize that our study contributes by highlighting the unique nuances and potential variations within this context. The integration of ST, given its interactive and multimedia elements, indeed holds promise in promoting students’ dedication and commitment to their academic tasks. However, the specific conditions, pedagogical approaches, and teacher roles play a significant role in determining the extent of this impact. Thus, our study adds valuable insights that contribute to the broader understanding of the intricate relationship between ST and AS among elementary EFL learners.

Regarding the Hypothesis on Academic Performance (AP):

The hypothesis driving this research asserts that the utilization of ST has a discernible impact on Academic Performance (AP) among elementary English as a Foreign Language (EFL) learners. AP encompasses students’ achievement in language proficiency, overall academic outcomes, and their ability to meet learning objectives, while ST encompasses a spectrum of digital tools and devices, such as tablets, smartphones, and interactive whiteboards, that offer new opportunities for engagement and interaction within the classroom.

Our findings concur with previous research conducted by [8,72–74] all of which have reported similar results regarding the effect of ST on AP. These studies collectively underscore the notion that the integration of ST has a tangible impact on students’ academic performance.

Moreover, a considerable body of research has delved into the impact of technology integration on AP, further substantiating our hypothesis. Notably, [75] investigated the influence of a mobile-assisted language learning program on the development of vocabulary among elementary EFL students. Their findings revealed significant improvements in students’ vocabulary knowledge, indicative of ST’s potential to enhance language proficiency.

Similarly, [76] explored the effects of computer-mediated communication on students’ oral proficiency in a language-learning context. Their results demonstrated notable enhancements in students’ speaking skills, further corroborating the positive influence of technology on AP.

ST, with its myriad features and functionalities, offers substantial potential to contribute to improved AP. Its interactive nature permits personalized and adaptive learning experiences, accommodating individual learning styles, preferences, and pace. Students can engage with multimedia resources, interactive quizzes, and educational apps, facilitating deeper understanding, critical thinking, and knowledge retention. Furthermore, by providing immediate feedback, scaffolding, and opportunities for self-assessment, ST can offer substantial support to students in their language learning journey and contribute to their overall AP.

Nevertheless, it is imperative to acknowledge potential challenges and constraints when considering the impact of ST on AP. Students’ varying levels of technological literacy, access to resources, and issues related to the digital divide may influence their ability to effectively harness ST tools. Moreover, the integration of ST should be thoughtfully aligned with pedagogical approaches and learning objectives to ensure that it complements and enhances classroom instruction rather than serving as a substitute for effective teaching practices.

The pivotal role of teachers is essential in maximizing the impact of ST on AP. Teachers can strategically design instructional activities that harness the capabilities of ST, offering guidance and scaffolding to support students’ learning. They can also monitor and assess students’ progress, providing timely feedback and supplementary support as needed. By synergizing the strengths of ST with effective teaching strategies, teachers can create a conducive learning environment that effectively promotes AP.

In conclusion, while our findings align with the existing research landscape, it’s essential to acknowledge that our study contributes by shedding light on the specific conditions and factors that underpin the relationship between ST and AP within the context of elementary EFL learners. The integration of ST indeed holds promise in enhancing students’ academic performance. However, the nuances and variations within this context, as well as the critical role of teachers, are essential aspects to consider, and our study offers valuable insights that contribute to a deeper understanding of this intricate relationship.

## Conclusion

This study explored the impact of ST on AE, AS, and AP among elementary English as a Foreign Language (EFL) learners. Our findings affirm the positive influence of ST on these key dimensions and delve into broader implications for teaching theory and pedagogical practices, all while recognizing the inherent constraints within our research. The study confirms that the integration of ST significantly enhances AE in elementary EFL learners. The multifaceted nature of ST, encompassing multimedia elements, interactive applications, and gamified learning platforms, fosters genuine eagerness for language learning activities. This aligns with contemporary teaching theories that emphasize active and learner-centered approaches, highlighting the transformative potential of ST in fostering a dynamic and engaging learning environment.

Furthermore, the investigation supports the hypothesis that ST positively influences AS among elementary EFL learners. Our study emphasizes the pivotal role of teachers, extending beyond existing research by highlighting the strategic design of instructional activities that harness the capabilities of ST. The interactive and multimedia features of ST not only capture students’ attention but also promote a sense of purpose and dedication to academic tasks, emphasizing the need for thoughtful integration aligned with curriculum objectives.

Consistent with existing literature, our study affirms that ST has a discernible impact on AP in elementary EFL learners. The implications for teaching theory become apparent as we recognize the potential of ST to contribute to improved language proficiency, critical thinking, and overall academic outcomes. The role of teachers is crucial in maximizing this impact, emphasizing strategic instructional design, timely feedback, and supplementary support to enhance students’ academic journey.

This study bridges the gap between theoretical underpinnings and practical implications, showcasing the transformative potential of ST in contemporary teaching. Our findings align with pedagogical theories that emphasize active learning, technology-enhanced instruction, and learner-centered approaches. Educators can leverage ST to create dynamic and engaging learning environments that capitalize on personalized and interactive experiences, aligning seamlessly with the evolving landscape of modern pedagogical practices.

While our study offers valuable insights, it is crucial to acknowledge the limitations inherent in any research endeavor. Generalizability may be constrained by contextual variations, including geography, culture, and educational settings. Disparities in technology access, familiarity, and external factors such as parental involvement may influence observed outcomes. Our research, while rigorous, recognizes these limitations, urging caution in extrapolating findings to diverse contexts.

In summation, this study not only sheds light on the intricate relationship between ST and AE, AS, and AP among elementary EFL learners but also provides a bridge between theoretical frameworks and practical applications in teaching. By acknowledging research constraints, we strive to present a nuanced understanding of the potential and limitations of ST in enhancing the educational experience of elementary EFL learners.

### Implications

Our findings strongly support the hypothesis that ST positively influences AE, AS, and AP among elementary EFL learners. Integrating ST into language learning activities kindles students’ motivation, enthusiasm, and interest in academic tasks, fostering eagerness for language learning. Furthermore, ST promotes AS by enabling self-directed learning, personalization, and collaboration, underpinned by technology’s adaptability and access to authentic resources.

### Pedagogical practices

Implications extend to teaching theory and pedagogical practices. The effective integration of ST aligns with contemporary teaching theories that emphasize active learning, technology-enhanced instruction, and learner-centered approaches. Educators can harness ST to create dynamic and engaging learning environments, capitalizing on personalized and interactive experiences. The findings underscore the transformative potential of ST in modern pedagogical practices.

## Limitations

Recognizing research constraints is essential. Generalizability may be limited due to potential contextual variations, including geography, culture, and educational settings. Disparities in technology access, familiarity, and external factors like parental involvement and classroom dynamics may influence observed outcomes. Additionally, while we employed rigorous methodology, the study acknowledges the limitations inherent in any research endeavor.

### Suggestions

To maximize the benefits of ST in the elementary EFL classroom:

Incorporate Interactive Learning Platforms: Utilize interactive learning platforms, such as gamified language learning apps and online discussion forums, to bolster engagement, motivation, AE, and seriousness.

Offer Customized Learning Experiences: Employ adaptive technology tools to provide tailored learning experiences, catering to individual needs, and promoting dedication to academic tasks.

Foster Collaborative Learning: Encourage collaborative learning through ST tools like virtual classrooms, enhancing peer interaction and cooperation, further fueling AE and seriousness.

In conclusion, this study illuminates the positive influence of ST on AE, AS, and AP among elementary EFL learners. It has direct implications for teaching theory and pedagogical practices, emphasizing the transformative role of ST in modern education. While acknowledging research constraints, we offer practical suggestions to harness ST’s potential fully.

## Supporting information

S1 File (SAV)
